# Genome survey sequencing and identification of genomic SSR markers for *Rhododendron micranthum*

**DOI:** 10.1042/BSR20200988

**Published:** 2020-06-18

**Authors:** Xiao-jun Zhou, Meng-xue Liu, Xiao-yu Lu, Shan-shan Sun, Yan-wei Cheng, Hui-yuan Ya

**Affiliations:** College of Life Science, Luoyang Normal University, 6 Jiqing Road, Luoyang 471934, China

**Keywords:** genomic characteristics, microsatellite molecular markers, next-generation sequencing, Rhododendron micranthum

## Abstract

*Rhododendron micranthum* is an evergreen shrub species widely distributed in China that has high ornamental and medicinal value. However, there is a lack of molecular and genomic data for this plant, which severely restricts the development of its relevant research. The objective of the present study was to conduct a first genomic survey of *R. micranthum* and determine its whole-genome sequencing scheme. Next-generation sequencing (Illumina Hi-Seq Xten) was used to measure the genome size of *R. micranthum*, K-mer analysis were employed to investigate its genomic profile. Finally, we conducted bioinformatics methods to performed SSR (simple sequence repeat) prediction based on the genomic data. The genome size of *R. micranthum* was estimated to be 554.22 Mb. The heterozygosity ratio was 0.93%, and the sequence repeat ratio was calculated to be 49.17%. The clean reads of *R. micranthum* were assembled into 2281551 scaffolds with a N50 value of 916 bp. A total of 479724 SSR molecular markers were identified in the *R. micranthum* genome, and 871656 pairs of primers designed for application. Among of them, 100 primer pairs were validated, and 71 primer pairs were successfully amplified. In summary, the *R. micranthum* genome is complex with high heterozygosity and low repeated sequences. In future whole-genome research in *R. micranthum*, higher-depth ‘2+3’ (Illumina+PacBio) sequencing may yield better assembly results.

## Introduction

**Figure 1:**
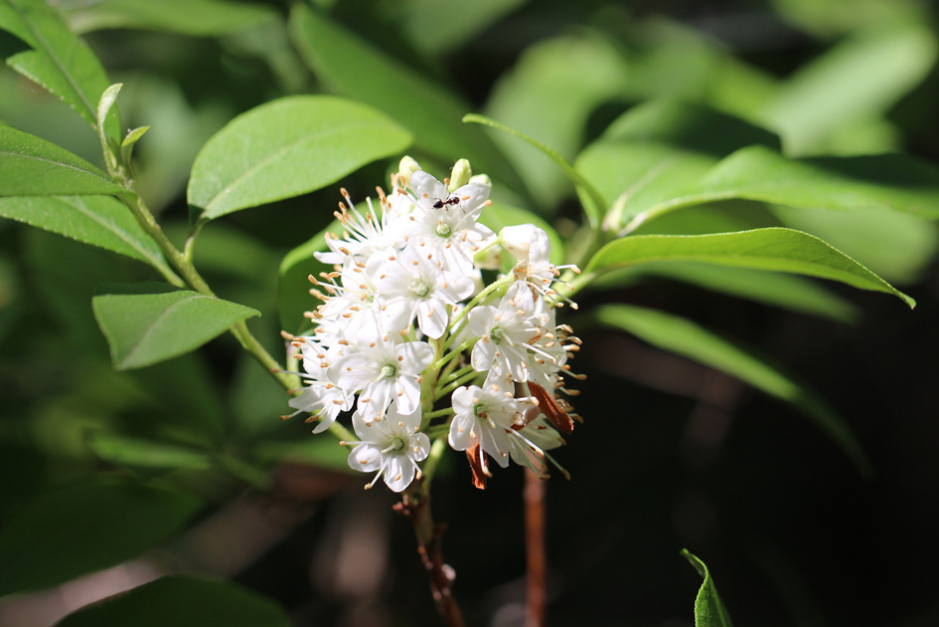
Leaves and inflorescences of *R. micranthum* in the Longyuwan National Forest Park of China (33°42′28″ N, 111°45′20″ E)

*Rhododendron micranthum* is an evergreen shrub species belonging to the genus *Rhododendron* in the family Ericaceae (Figure 1). It is widely distributed in China, growing on the hillsides of thickets, valleys, cliffs, and rocks at an elevation of 1000–3000 m [1]. Compared with other rhododendrons, *R. micranthum* not only has strong ornamental value, but also has high medicinal value [2]. However, the current research in *R. micranthum* mainly focuses on chemical composition analysis and artificial cultivation [3–5], and molecular-level research has not yet been carried out.

Genome contains all the information of controlling biological characters and ultimately determines the transmission of genetic material. The genomic content, also known as the *C* value, refers to the DNA content of the haploid nucleus of a species. More and more studies have found that biological genome information is related to physiological functions [6]. To explore the genes related to the effective components and excellent agronomic characters of plants, and analyze the metabolic pathway and regulatory mechanism from the genome-wide level can lay the foundation for the improvement of medicinal plant varieties and the protection of genetic resources [7]. In the present study, Illumina Hi-Seq technology combined with K-mer analysis was used to evaluate the genome size and characteristics of *R. micranthum*, which laid the foundation for fine sequencing of the whole genome and will be conducive to further understand its pharmacological effect. SSR (simple sequence repeat) molecular markers have the characteristics of abundant quantity, high polymorphism, co-dominance, and easy automatic analysis. The SSRs developed based on *R. micranthum* genomic survey are helpful to germplasm resources identification, genetic breeding and provides the molecular basis for the conservation and utilization of its gene resources.

## Materials and methods

### Plant materials and genome sequencing

Leaf samples from a single individual *R. micranthum* were collected from Longyuwan National Forest Park in Luanchuan Country. After disinfection and cleaning with 70% alcohol, leaves were subjected to the total genomic DNA isolation by the CTAB (cetyltrimethylammonium bromide) method [8]. The DNA were electrophoresed on 1% agarose gel electrophoresis and the samples were subsequently quantified by a 2100 Bioanalyzer system (Exon Biotech).

The *R. micranthum* DNA samples of suitable quality were randomly sheared into 300–350 bp fragments using an ultrasonicator (Covaris Inc, U.S.A.). Electrophoresis was used to recover the DNA fragments of required lengths before end-repair, following with poly A-tail and sequencing adapters were added. The obtained fragments with a length of ∼350 bp were used to construct two paired-end DNA libraries, and then sequenced with a read length of 2 × 150 bp using the Illumina HiSeq XTen platform, according to the manufacturer’s protocol. After we filtered reads that would interfere with subsequent information, reads with an *N* (unable to determine base information) ratio greater than 10%, reads with adapters, duplicated reads caused by PCR amplification, and low-quality reads from the raw reads, clean reads underlying all following analyses were acquired. High-quality Illumina sequencing reads were submitted to the NCBI Short Read Archive (accession number: PRJNA613232).

### Genome size estimation and genome survey

The clean data with high-quality reads were used for K-mer analysis. Based on K-mer (k = 17) frequency distributions, we used GENOMESCOPE to estimate the characteristics of the genome (genome size, repeat content and heterozygosity rate) [9]. The filtered reads were aligned to assembled sequence using SOAP to obtain the base depth. The 10 kb non-overlapping sliding windows along the assembled sequence were used to calculate GC content and sequencing depth [10].

### Analysis of SSR molecular markers

The assembled genome sequences were searched for microsatellite loci using MIcroSAtellite identification tool (MISA) program [11,12]. The parameters were set based on the minimum number of iterations: 4 repeat units for hexa-, penta-, and tetranucelotides, 5 for trinucleotides, 6 for dinucleotides, and 10 for mononucleotides. Primer pairs were designed using the Primer 3 software in the flanking regions of SSRs [13]. To validate the designed primers, a total of 100 primer pairs that motif length more than 20 bp were synthesized and PCR was carried out for amplification. The PCR procedure was carried out in 10 μl volume containing 20 ng of genomic DNA, 0.25 μM of forward and reverse primers and 5 μl of 2 × PCR Mixture (Tiangen, Beijing, China) with conditions as follows: denaturation for 5 min at 94°C followed by 30 cycles of 50 s at 94°C, 45 s for annealing at 60°C and 1 min at 72°C, and a final extension at 72°C for 10 min. Amplification products were resolved by 10% PAGE (polyacrylamide gel electrophoresis) and visualized by silver staining. The size of each SSR-PCR products was determined in comparison with pBR322 DNA/MspI marker (Tiangen, Beijing, China) [14].

## Results and discussion

### Sequencing and quality evaluation of *R. micranthum*

**Figure 2:**
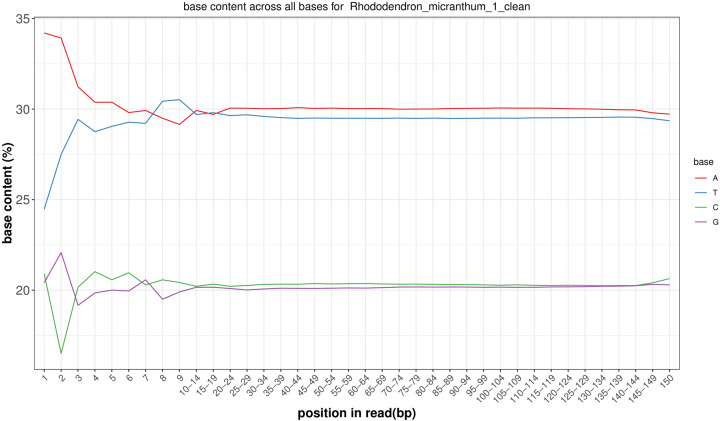
Distribution figure of AT and GC content

After filtering and correction, a total of 60.05 Gb of *R. micranthum* clean bases were obtained with the Q20 and Q30 values were 96.6% and 90.9% respectively. Figure 2 shows the proportion of single bases, which is used to detect whether AT and GC separation is present. It can be seen that the content of A and G and C and T are close. The results demonstrated that the sequencing quality was good. We randomly selected 1000 clean reads as a query sequence with BLAST (The Basic Local Alignment Search Tool) against the NT (Nucleotide Sequence Database) from the NCBI (National Center for Biotechnology Information), the top five species for comparison are *Rhododendron simsii* (1.43%), *Camellia sinensis* (1.32%), *Vaccinium macrocarpon* (0.69%), *Vitis vinifera* (0.26%), and *Empetrum rubrum* (0.18%). The results showed that there was no contamination from other species.

### Genome size estimation by K-mer analysis

**Figure 3:**
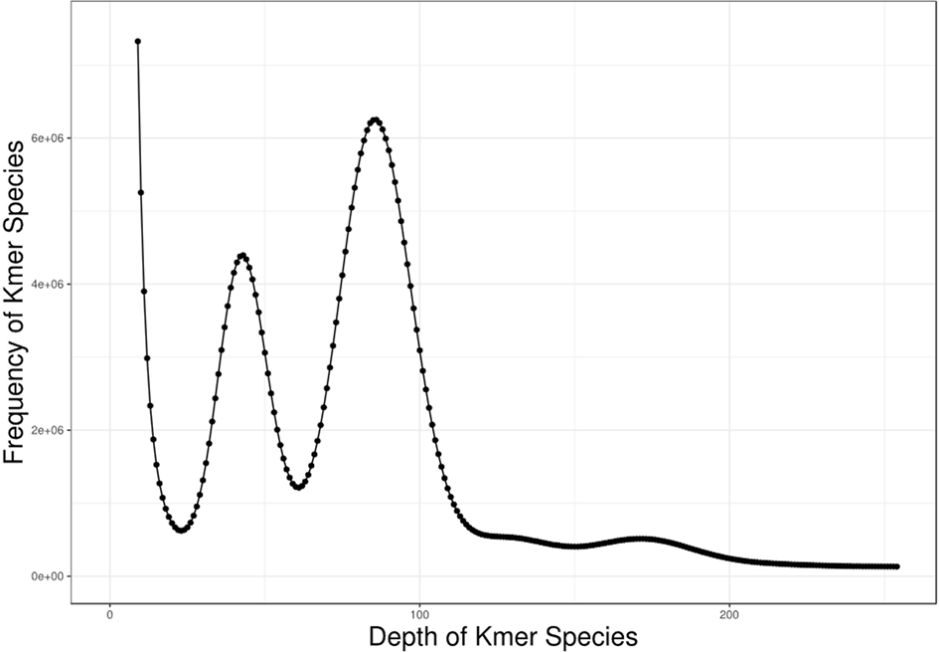
Distribution curve of K-mer (k = 17) of *R. micranthum*

All the clean data were performed on K-mer analysis. For the 17-mer frequency distribution (Figure 3), the number of K-mers was 49015508228 and the peak depth distribution was set at 86×. The estimated genome size was 570.17 Mb, which was calculated via the following formula: Genome size = K-mer num/K-mer depth [15]. Then, the genome size was reformulated by excluding the K-mer error, and the revised genome size was 554.22Mb. After computed the proportion of heterozygous sites in the sequence, we obtained a gene heterozygosity ratio of 0.93%. By calculating the percentage of 1.8-times the number of K-mers after the main peak over the total number of K-mers, we obtained the genome sequence repeat ratio percentage for *R. micranthum* was 49.17% (Table 1).

**Table 1:** Genome estimation based on K-mer statistics

Sample	K-mer number	K-mer depth	Genome size (Mb)	Revised genome size (Mb)	Heterozygous ratio (%)	Repeat (%)
*R. micranthum*	49015508228	86×	570.17	554.22	0.93	49.17

Genome survey gives a preliminary understanding of the genomic characteristics before large-scale genome sequencing for one species. Genome size, is usually described using the *C*-value, refers to the DNA content of all biological haploids. The *C*-value shows species-specific characteristics and is constant for every species [16]. The genome size is related to different biological parameters, such as cell size and cell cycle, economic characteristics, and stress resistance. And studies have found that genome size plays an important role in plant evolution and adaptation [17]. K-mer estimation of genome survey was successfully applied in the prediction of genome sizes for many non-model plant species, such as *Apocynum venetum* [17], *Betula platyphylla* [18], *Ammopiptanthus mongolicus* [19], and *Liriodendron chinense* [20]. There is great variation in genome sizes among angiosperms, the largest genome species is *Paris japonica*, with a genome size of 150 Gb [21], while the smallest genome species is *Genlisea tuberosa*, with a genome size of 61 Mb [22]. In the present study, The genome size of *R. micranthum* was close to the size of *R. indicum* (567 Mb) [23], but smaller than *R. simsii* (608 Mb) [23], *R. delavayi* (695.09 Mb) [24], and *R. williamsianum* (650.8 Mb) [25]. The genome survey of *R. micranthum* has provided a solid foundation for its whole-genome sequencing, and promote the exploration of its medicinal value.

The genome of plants can be divided into high heterozygosity (heterozygosity ratio ≥0.8%) and low heterozygosity (0.5% ≤ heterozygosity ratio <0.8%), low repetitive genomes (repeated ratio <50%) and highly repetitive genome (repeated ratio ≥50%) [26]. This preliminary analysis determined the *R. micranthum* genome to have heterozygosity ratio of 0.93%. K-mer distribution curve obtained repeated sequence percentage of 49.17%. Thus, *R. micranthum* genome is complex with high heterozygosity and low repeated sequences. It is difficult to assemble a genome if the heterozygosity rate is higher than 0.5% [15]. The characteristics of the *R. micranthum* genome might impact the accuracy of genome size estimation. This was the reason that revision of the genome size was performed. Repeated sequences are one of the major factors that control the recombination and regulation of structural genes [17], and the further investigation is required to understand the functions of repeated sequences in *R. micranthum*.

### Genome assembly and GC content analysis

The clean reads were used for *de novo* genome assembly of *R. micranthum*, and a K-mer value of 41 was selected to construct the contig and scaffold. A total of 757117087 bp scaffolds were derived, with a N50 scaffold value of 916 bp (Table 2). The N50/N90 of the contigs/scaffolds were derived by ordering all sequences, adding all the contigs/scaffolds from the longest to the shortest and when the added length reached 50%/90% of the total length of all contigs/scaffolds, the length of the last added contig/scaffold was the N50/N90 [27].

**Table 2:** Statistics of assembled genome sequences for *R. micranthum*

	Total length (bp)	Total number	Total number (≥2 kb)	Max length (bp)	N50 (bp)	N90 (bp)
Contig	845872500	2307871	65029	46257	794	114
Scaffold	757117087	2281551	61736	46157	916	124

**Figure 4:**
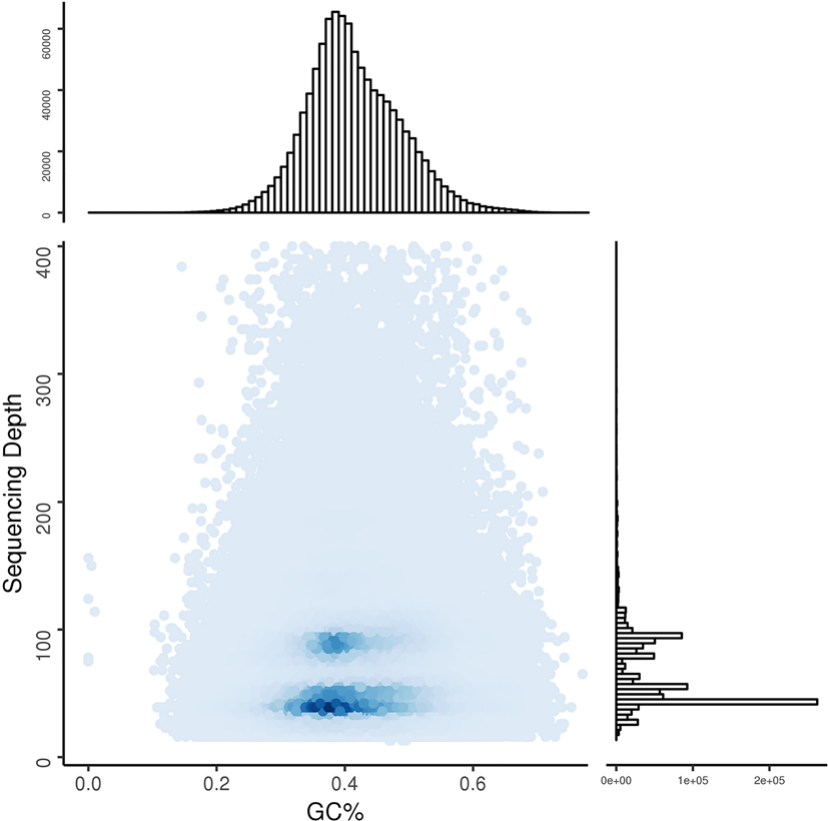
Average sequencing depth and GC content of *R. micranthum* genome data For the bar graphs, the *x*-axis is sequencing depth distribution and the *y*-axis is GC content distribution. For the spot graphs, the *x*-axis is GC content and the *y*-axis is sequencing depth.

The average sequencing depth and GC content of the *R. micranthum* genome were plotted along the assembled contigs which length more than 200 bp (Figure 4). The density points were only concentrated in the 30–50% range, and the average GC content was 40.4%. Shangguan et al. summarized that GC contents of plant genomes mostly range within 30–47% [28]. The GC content of the *R. micranthum* genome is 40.4%, which is like *R. simsii* and *R. indicum* (39%) [23]. It is higher than that of *Medicago sativa* and *Malus pumila*, which have a GC content of less than 30%, but is lower than that of *Sorghum bicolor, Zea mays*, and *Oryza sativa* which have a GC content of more than 40% [16].

### SSR identification

Recently, SSRs consist of 1–6 motifs are utilized as molecular marker in many studies of genetic maps and genetic populations [29]. The repetitive sequence of genomic DNA not only affects the advanced structure of chromosomes but is also very useful in genome evolution and rearrangement [30,31]. The assembled contigs of *R. micranthum* were searched for SSR markers by using the MISA software (http://pgrc.ipk-gatersleben.de/misa/misa.html). A total of 479,724 SSRs were identified. Among these SSRs, the mononucleotide was the most abundant SSR marker, accounting for 54.47% (261,325) of the total SSR markers, which was followed by di- (189,454, 39.39%), tri- (24,369, 5.08%), tetra- (2675, 0.56%), penta- (1043, 0.22%), and hexanucleotide (858, 0.18%) SSRs (Table 3). There was a large proportion of both mononucleotide and dinucleotide SSR markers which amounted to 93.86%.

**Table 3:** Characteristics of SSR markers for *R. micranthum* genome

Repeat number	SSR type						
	Mononucleotide	Dinucleotide	Trinucleotide	Tetranucleotide	Pentanucleotide	Hexanucleotide	Total
5	–	–	13681	1797	808	557	16843
6	–	40777	5189	554	174	167	46861
7	–	27838	2462	197	45	66	30608
8	–	21967	1172	70	11	27	23247
9	–	17040	679	32	3	12	17766
10	86706	13808	445	7	–	5	100971
11	49577	11103	256	10	1	5	60952
12	32902	9324	158	4	–	6	42394
13	23281	8297	118	–	–	1	31697
14	17659	7726	68	–	–	–	25453
15	14670	6649	37	–	–	3	21359
≥16	36530	24925	104	4	–	9	61572
Total	261325	189454	24369	2675	1043	858	479724

**Figure 5:**
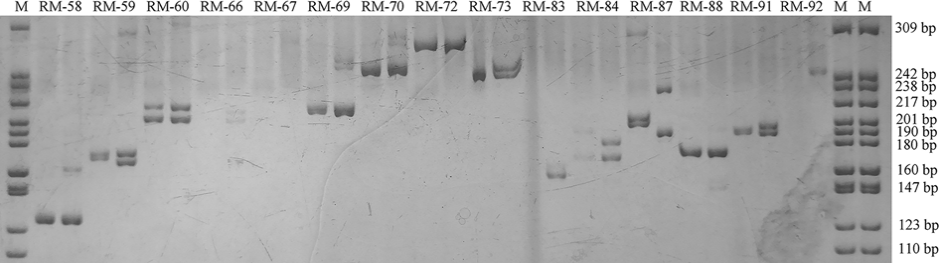
SSR-PCR amplification profile of *R. micranthum* M is the DNA marker pBR322 DNA/MspI. RM-58, RM-59, RM-60, etc. are primer numbers.

Among the mononucleotide repeats, A/T ratios were greater than the G/C ratio, accounting for 98.17% of the total number of repeat units. Among the dinucleotide repeats, the number of GA/TC repeats was the highest (73194), accounting for 38.63%, followed by AT/TA repeats (28775, 15.19%), while CG/GC repeats were lowest (912), accounting for only 0.48% of the total number of repeats. Among the trinucleotides, the number of CAC/GTG repeats was the highest (2932), accounting for 12.03% of the total. In the present study, a total of 290552 sequences that include SSRs suit to designed primers. Three pairs of primers were designed for each sequence and 871656 pairs of primers designed for application. To validate the SSR markers, 100 primer pairs were synthesized and tested for PCR amplification, and 71 primer pairs were successfully amplified and exhibited expected sizes (Figure 5). Information on the 71 pairs of primers was shown in Supplementary Table S1.

A large number of genome-wide SSR loci have provided a foundation for the further construction of high-density genetic maps and the study of the regulatory mechanisms of medicinal ingredients for *R. micranthum*. This also provides a good reference for molecular marker and future genome research.

## Conclusion

This is the first study to measure the size of the *R. micranthum* genome and preliminarily assess the corresponding parameters. The genomic characteristics of *R. micranthum* include a genome size of 554.22 Mbp, a heterozygosis rate of 0.93%, and a repeat rate of 49.17%. A total of 479724 SSR markers were developed from the *R. micranthum* genome data and 871656 pairs of primers were designed. These results and dataset may provide a new resource for future genomic analysis, molecular breeding, and key genes analysis for effective components synthesis in *R. micranthum*.

## Supplementary Material

Supplementary Table S1

## Abbreviations

MISA: MIcroSAtellite
SSR: simple sequence repeat
