# Supplementary material for: Genome survey sequencing and identification of genomic SSR markers for Rhododendron micranthum
Source: Biosci Rep. 2020 Jun 18;40(6):BSR20200988. doi: 10.1042/BSR20200988 (PMC7303352; doi:10.1042/BSR20200988)
Supplement: Supplementary Table S1 [file BSR-2020-0988_supp.pdf]

Table S1 Information of the 71 pairs of SSR primers in*R.micranthum*

Primer No.	Primer sequence (5'→3')	Motif	Expected size (bp)	Ta (℃)
RM-1	F:ACCTCACCCCTTCCTCAACT R:CGTCAAATGGGAAGAAAGGA	(TTC)9	218	55
RM-2	F:GGATCCCCATCTTTTTCTTCA R:GATTCCTCCTCGAGCAACAG	(TCA)9	193	54
RM-4	F:GAAGGTCCGCATTTTAGCAA R:CTCACCCAGGTTGTATTCCG	(ATT)9	169	54
RM-6	F:CATGAAGGCAATTAGCCAAAA R:TCTGAAACATACCGAAGGGG	(AAG)9	272	53
RM-7	F:CAATCCACCACATGACGAAG R:GGGTTATGGGAACAGAAGCA	(ACC)9	240	54
RM-8	F:GGGAACCCCTCTGTTGAAAT R:GAAACCCTAACCGGTCCTTC	(AAG)9	248	55
RM-9	F:AGTTGGCCGTTCATAAGGTG R:TGGCAATTGCTATTGATGGA	(ACA)9	141	53
RM-10	F:GGCAAGAGTGGCATGAAAGT R:TGAAGGATTGCAAATTGTCCT	(CTT)9	136	54
RM-15	F:TCAATCATCGTCTGCCCATA R:GGTATGTCAACAACGGCAAA	(TGT)9	272	53
RM-16	F:GTGTTGGAATGGGTCACGTA R:CACCTCGGCCCTAACTATTG	(GAA)9	175	55
RM-17	F:CTTGATCATTTGGCCCACTC R:GTGACCTTCCACCAAGCAAT	(TTA)9	176	55
RM-18	F:TATTGCCCCTTTTGCTTTTG R:GAATTTGGGGATGAGGTGAA	(CAG)9	129	53
RM-19	F:TACAATGCCGTCTTCTGCTG R:TTGTGAGAAGGAAAAACCCG	(CCG)9	177	54
RM-20	F:CCGTTCCTCGCTCGTTTTAT R:TTGGCAATGCTAGCCTTTTT	(AAT)9	279	54
RM-22	F:TTAAACCCTTATGCGGCAAC R:CACAACGGTGAAAACCAGTG	(CCT)9	210	54
RM-23	F:CCCGGGACCAAGTTTTTATT R:CGTCAAAAAGCGGAGAGTTC	(TTA)9	259	53
RM-24	F:TTCGCCTACCTTCCAATGTC R:CTTCACAACCACCACCACAG	(AAG)9	165	56
RM-25	F:ACCAACGAAATGATCAAGGC R:CGTGACAAGTGGTATCCGTG	(TTA)9	188	55
RM-26	F:TGAAGGGCATTTAAGAACCG R:AGTGGTGGGACTCGTACAGC	(ACC)9	215	56
RM-27	F:CTTTGGGTTTCGACTTGAGG R:TCGATACCAGGTCAGTTCGAC	(TTG)9	168	55
RM-28	F:CTTCTTTCTGGCTTGGCTTGR:TAAGCTGGAGGAGGAGGTCA	(TAC)9	161	56
RM-31	F:GGTTCAGATTTTCGTTCCGA R:GCGATAACATGATGCCGAAT	(TAA)9	271	53
RM-32	F:GAACAGAACGGAGCTCCAAC R:GGAAAGGAAAATTCGTGGAA	(AAG)9	279	53
RM-33	F:CGTGCACCAAAATTGGAGAT R:CAAACTTGTGGTAATGCATCC	(TTC)9	182	53

RM-37	F:ACATGCATGCAAAACTCCCT R:TGCCTTCTCCAGAGCAGATT	(ATA)9	242	55
RM-38	F:CGTGATGGCTATTGAAGGCT R:ATCCGTTTGATGTATGGGGA	(TTC)9	206	54
RM-39	F:GCGGAGACGGTGTGTTTATT R:GCCTCCTTTTCCACCTTCTC	(GGA)9	185	56
RM-40	F:AGGCTGAAGAGACGAGGTCA R:CTGGGAGTTCTGCCCAATTT	(AGA)9	189	57
RM-41	F:GGAGAAAGCGAAGAACGAGA R:CCCATCATCCTCCTCAATGT	(AAC)9	196	54
RM-42	F:CGAGAGATTGATAGAGGGCG R:AGTGAGAGAGCGACGGAGAG	(CTT)9	269	57
RM-44	F:AACCAATTGGCAAAGAGTGG R:AAAAATTTGGTCAAACGGAAA	(AAG)9	278	51
RM-45	F:GGGTTCCAAAGTTCGACAAA R:TTACATGACCTTCCCCCAAA	(GTT)9	204	53
RM-47	F:TGTTTGGAACATGGGAAAAA R:CGCAAACTTATGGAATGCAA	(AAG)9	270	51
RM-48	F:TCGAACAAAACATCCGTCAA R:GCATTTGGAATTTGCGTTTT	(CAC)9	278	51
RM-49	F:TGGTGCTAGGAATGATTGGA R:GAGCGAGTAATATCCAGCGG	(AAG)9	257	54
RM-51	F:GGCGCAACTTAAGAAAACCA R:CGAACGATCATAACACCCCT	(AGT)9	263	54
RM-53	F:TCTCGTTAATGAAGGCTGGC R:TCCCATCATCAAGCCATACA	(ATT)9	193	54
RM-54	F:ATGTGTCGCTGAGTGAGTGG R:AGTGACCTTTCGGGGTCTTT	(TAT)9	203	57
RM-57	F:TTTCCGTGTTTGGGTTTCTC R:TCAGGTGAATCGGATGACAA	(CTT)9	269	53
RM-59	F:GAAGATCGATCGAGGTCCAA R:TAGTCACTGCCAAAACAGCG	(TCT)9	158	55
RM-60	F:ATATGCCTATCTCCACCCCC R:AAATTGTCTTCGTTGCTGCC	(CTC)9	210	55
RM-61	F:AGATGTAGGCGGGTGAATTG R:ACAAGGCTACCCCTGTCTCA	(TCC)9	192	57
RM-63	F:GTGCTAGCATTGCCAACAGAR:CCTAACCCGGAAAGGAAAAA	(TCC)9	115	54
RM-64	F:CGGAACAAAAAGAAGTGGGA R:AAGATGGCATTGTCCGTTTC	(AGG)8	106	53
RM-68	F:ACGAAGGCCCTACTTCCTGT R:GGGTGGTCATGTTCCATTTC	(AAG)8	209	56
RM-69	F:AGGAATCGGTCGGAAGAGTT R:ACGCCTTCATCTTCGTCATC	(GAT)8	204	55
RM-70	F:CACACTGGATTGTGGTGGAG R:GAGGACAAATTTACGCCCAA	(AAG)8	243	55
RM-72	F:GGAGGAAAGAAGGGAGTTGG R:GAGGCGAAAGATACCAGCAA	(CAC)8	277	55
RM-73	F:ACGAGCCCGACAATTGTAAC R:CAGGAAGAAATGGAGGGTGA	(TTC)8	231	55
RM-74	F:CACACACATCGGTTCTTCGT R:CACAGCAAGAGGGTTGGTTT	(ACA)8	236	56

RM-75	F:GTTCCCATAGCCCCAATGAT R:CGCAGGATTCGAGTGTAGGT	(TTG)8	139	56
RM-76	F:TCTTCATAAACGCCACCACA R:AATGCACTCCCTCATTTTCG	(CTC)8	108	53
RM-77	F:TTGGTCGCTTATTTGGAAGG R:TGGTTTTTACGTTGCCCATT	(TAG)8	214	52
RM-81	F:GGTTGGCAAACTCCCTATCC R:TTAGCGGGCAAGAAGAAAAA	(ATT)8	173	54
RM-82	F:CATAGGGCCCAATCCTTTTT R:GTGAAAGCTGTGGCCAAAAT	(CCA)8	221	53
RM-83	F:CCTTCCCTTTCTTCCCAAAG R:AAGTCATGAGGGTCTCGTGG	(CCA)8	157	55
RM-84	F:ATGGTTTCAAGGAGTGGACG R:CGGAAAGAGAGAAGCACCAG	(TGC)8	165	55
RM-85	F:CGGCTAGGTGTAAATGGGAA R:AAAGGGGGAATTTGACAAAAA	(AAG)8	253	52
RM-86	F:GGTGATGGTTGTTGCTGTTG R:TGAAGCAAATGCCAGCTATG	(TGC)8	277	54
RM-87	F:GGCAGAAAATACGCTTGCTC R:ACCGAGTTTTGTGGAGGTTG	(CAG)8	192	55
RM-88	F:CATCCCCCTTCTCTCTTTCC R:AATTTGGTGGTGTGCTGACA	(TTC)8	170	55
RM-89	F:ACACGAGAGGGTGACAGCTC R:CCCCTTTTCAATCCTCCAAT	(TAC)8	276	56
RM-91	F:GCATGACAAAAAGGGAATCG R:TTAGCTAGGCTGGTGGAGGA	(TAG)8	185	55
RM-93	F:TGTGTGGCTCACATTTTCA R:TGGTGGATATTTCACAAACATGA	(AGT)8	159	52
RM-94	F:ATCCTTTGAGGGGAAGGAGA R:CAGCCCAAATCCTACTCAGC	(TTC)8	250	56
RM-95	F:CAAGGGTTACCAAACACTGC R:AAGGGGATCCGTTTAATTCG	(AAG)8	220	53
RM-96	F:CCTCCCAATTGACTGACGAT R:TGACGTGATGTTGCATTTTG	(CCA)8	280	53
RM-97	F:TAAAAACAAAACCGCGCAAT R:AACATCCCTAACGGTATGCG	(AAT)8	239	53
RM-98	F:CTCCATTTGCCTTTTTCTCG R:AAAGCACAGGAGGGTGAAGA	(ATC)8	233	54
RM-99	F:GCAAGAAAAGCCCTGAGAAA R:TCTAGCATTGGGGATGAAGG	(CTT)8	138	54
RM-100	F:TAGCCCAATCGTCTCCAATC R:ACTCTCCCTCAGTTGCCTCA	(GGA)8	257	56
